# Fast 2D Laser-Induced Fluorescence Spectroscopy Mapping of Rare Earth Elements in Rock Samples

**DOI:** 10.3390/s19102219

**Published:** 2019-05-14

**Authors:** Peter Seidel, Sandra Lorenz, Thomas Heinig, Robert Zimmermann, René Booysen, Jan Beyer, Johannes Heitmann, Richard Gloaguen

**Affiliations:** 1 Helmholtz-Zentrum Dresden-Rossendorf, Helmholtz Institute Freiberg for Resource Technology, Chemnitzer Str. 40, 09599 Freiberg, Germany; s.lorenz@hzdr.de (S.L.); t.heinig@hzdr.de (T.H.); r.zimmermann@hzdr.de (R.Z.); r.booysen@hzdr.de (R.B.); r.gloaguen@hzdr.de (R.G.); 2 Technische Universität Bergakademie Freiberg, Institute of Applied Physics, Leipziger Straße 23, 09599 Freiberg, Germany; jan.beyer@physik.tu-freiberg.de (J.B.); johannes.heitmann@physik.tu-freiberg.de (J.H.); 3 School of Geosciences, University of the Witwatersrand, Johannesburg 2000, South Africa

**Keywords:** laser-induced fluorescence, rare earth elements, imaging sensor, optical spectroscopy, reflectance spectroscopy

## Abstract

Due to the rapidly increasing use of energy-efficient technologies, the need for complex materials containing rare earth elements (REEs) is steadily growing. The high demand for REEs requires the exploration of new mineral deposits of these valuable elements, as recovery by recycling is still very low. Easy-to-deploy sensor technologies featuring high sensitivity to REEs are required to overcome limitations by traditional techniques, such as X-ray fluorescence. We demonstrate the ability of laser-induced fluorescence (LIF) to detect REEs rapidly in relevant geological samples. We introduce two-dimensional LIF mapping to scan rock samples from two Namibian REE deposits and cross-validate the obtained results by employing mineral liberation analysis (MLA) and hyperspectral imaging (HSI). Technique-specific parameters, such as acquisition speed, spatial resolution, and detection limits, are discussed and compared to established analysis methods. We also focus on the attribution of REE occurrences to mineralogical features, which may be helpful for the further geological interpretation of a deposit. This study sets the basis for the development of a combined mapping sensor for HSI and 2D LIF measurements, which could be used for drill-core logging in REE exploration, as well as in recovery plants.

## 1. Introduction

The rise of modern high-technology industries, such as semiconductor manufacturing or automotive engineering, has been accompanied by an increased complexity of products, which now include a great variety of elements. Many of the new material components designed during the last decades, such as light-emitting diodes, permanent magnets, and catalysts, contain large amounts of rare-earth elements (REE), a group of 17 metals comprising the lanthanoid group, scandium, and yttrium. The increasing demand for REEs, reaching a global production of 130,000 tons in 2017 [1], led to extended exploration and mining activities over the last decade. Despite their name, REEs are not “rare” in absolute number, but are rarely concentrated and mostly occur homogeneously and are usually finely disseminated in their host rocks. The low relative abundance, combined with a heterogeneous distribution of potential ore bodies on a global scale, makes exploration for REEs challenging. For larger exploration campaigns, many kilometers of drill cores are extracted and selected rock pieces are analyzed by geochemical methods, such as mass spectrometry or electron microscopy. These techniques are time- and cost-consuming, destructive, and can only be performed for small segments of the entire drill core. To overcome these obstacles, several non-destructive alternative techniques, which are fast and can be used for in-line analysis, have been tested for core logging. These techniques include X-ray fluorescence [2], laser-induced breakdown spectroscopy [3], and optical reflectance spectroscopy [4].

The latter, also known as hyperspectral imaging (HSI), has proven capable both for indirect remote sensing of REEs by identifying their host rocks using space and airborne detectors [5], and for the direct identification of REEs in drill cores when the distance between sample and detector shrinks to cm-m scale [6]. The good sensitivity in sub-% range for several REEs such as neodymium (Nd) and dysprosium (Dy) [7], together with high acquisition speed and robustness, promoted the introduction of commercially available core logging tools based on reflectance spectroscopy. However, another opportunity to identify REEs selectively and sensitively is laser-induced fluorescence spectroscopy (LIF). This technique has been employed extensively in the last two decades for REE detection in synthetic crystals [8] and various minerals (see overview in [9]). 

We recently started to investigate the potential benefits of combining hyperspectral imaging and laser-induced fluorescence to profit from the advantages of both techniques, such as their easy scalability, high acquisition speed, and high sensitivity for LIF [10,11]. Based on the idea of developing a new sensor, which is capable of identifying REEs in complex rocks, we pursue a systematic approach to extend our knowledge on the applicability of the LIF-HSI combination. In a first fundamental study, the signatures of synthetic REE phosphates and fluorides for reflectance and laser-induced fluorescence spectroscopy were identified and correlated to physical processes within these model substances, extending the spectral database for further studies [9]. In a second step, we investigated the spectra of REE-bearing mineral grains from different deposits all over the world [10], building up a database for both HSI and LIF spectra for the most abundant REE minerals. The increasing complexity of the materials (e.g., mixed REE salts with variable stoichiometry) led to the appearance and extinction of several absorption and luminescence peaks, which complicated the interpretation of obtained spectra. However, a combination of the HSI and LIF techniques proved to be beneficial for the identification of rare earth elements in these materials.

These studies focused mainly on spatially homogeneous, small samples with high amounts of REEs (up to 60 wt%). In this article, we aim for the next step towards a potential sensor for REE detection in drill cores, investigating common rock samples from current exploration areas with a combination of reflectance and laser-induced fluorescence spectroscopy. The emphasis of this study is placed on the development of surface mapping by LIF. When using rocks, several new constraints have to be taken into account: the relative amount of REE-containing phases in the low- or even sub-% range, the complex host rock matrices, and the high spatial heterogeneity, in particular for finely grained samples. Thus, a careful interpretation of obtained spectra with respect to signal position and shape is needed, based on the findings from the previous studies on REE salts and minerals. In addition, scanning electron microcopy-based X-ray mapping (known as mineral liberation analysis, or MLA) is employed to validate the findings from the HSI and LIF maps and to correlate the detected REE areas to the mineral phases.

## 2. Materials and Methods

We collected several rock specimens from one South African and three Namibian deposits, which were prepared in our labs to ensure a fitting sample size for our spectroscopy experiments. For several samples, the surfaces were especially smoothed to compare them to unprepared surfaces. The non-invasive techniques (HSI and LIF) were performed before the MLA measurements, due to the latter changing the state of the surface irreversibly.

### 2.1. Rock Sampling and Preparation

From a collection of 13 rock samples, we used two rocks from two Namibian deposits, Lofdal Farm and Epembe, for the feasibility tests of 2D LIF mapping for REE detection in rocks.

The Lofdal Alkaline Carbonatite Complex in central Namibia is a well-studied REE deposit [12,13,14], exhibiting a high enrichment of heavy rare earth elements (HREE, Eu–Lu) compared to light rare earth elements (LREE, La–Sm). Thus, rock samples from this deposit show high concentrations in a broad range of lanthanides, making it ideal to test 2D LIF mapping. In the Epembe deposit, critical elements (Nb, Ta, P, LREE) are hosted within two high-grade zones of hydrothermal origin [15,16]. The REE pattern is dominated by LREE and mineralization is hosted by (in order of importance) REE-rich apatite, monazite, and secondary REE-fluorocarbonates. All rock samples were collected during a field campaign, where most promising sampling spots had been identified by preliminary remote sensing observations of the area, combined with a detailed survey on the ground. These samples were analyzed by conventional whole-rock techniques, such as X-ray diffraction, X-ray fluorescence, and inductively-coupled plasma mass spectrometry in a laboratory environment. The setups and parameters used for these methods are described in more detail in [17].

**Figure 1:**
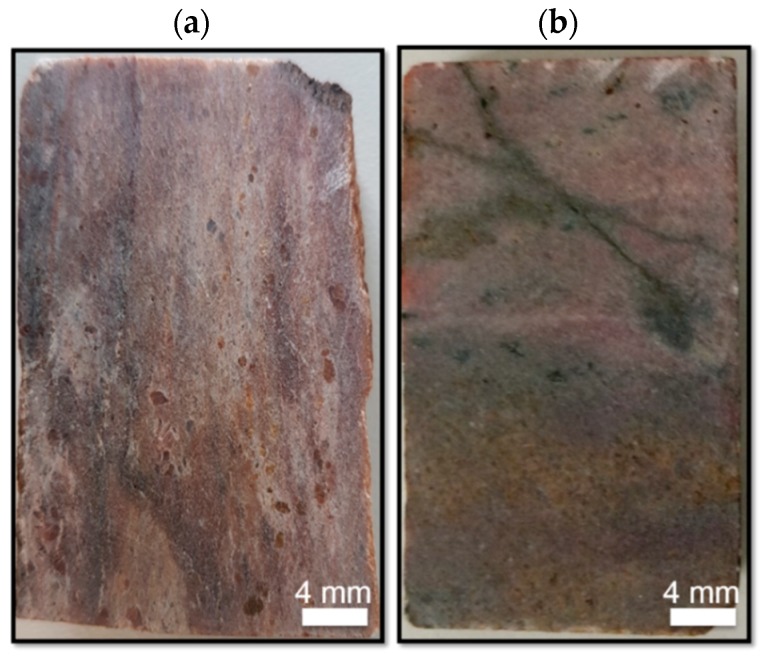
RGB images of the surface from the 40 × 20 mm^2^ large rock pieces from the rare earth element (REE) deposits: (**a**) Epembe (NA-RZ-05); (**b**) Lofdal Farm (NA-RB-02).

To obtain a planar surface, the collected rock specimens were cut and then sliced into 10 mm thick rock pieces, which were subsequently gritted to obtain a low surface roughness of approximately 15 µm (Figure 1). For high-resolution MLA measurements, the rock pieces were further gritted, fixed onto a glass substrate, and finally lapped and polished until a surface roughness of about 0.25 µm was reached. Importantly, the thickness of the rock piece was kept higher than 1 mm. Otherwise, some parts of the sample became partially transparent to visible light, which distorts HSI and LIF analyses.

### 2.2. Laser System and Data Processing

**Figure 2:**
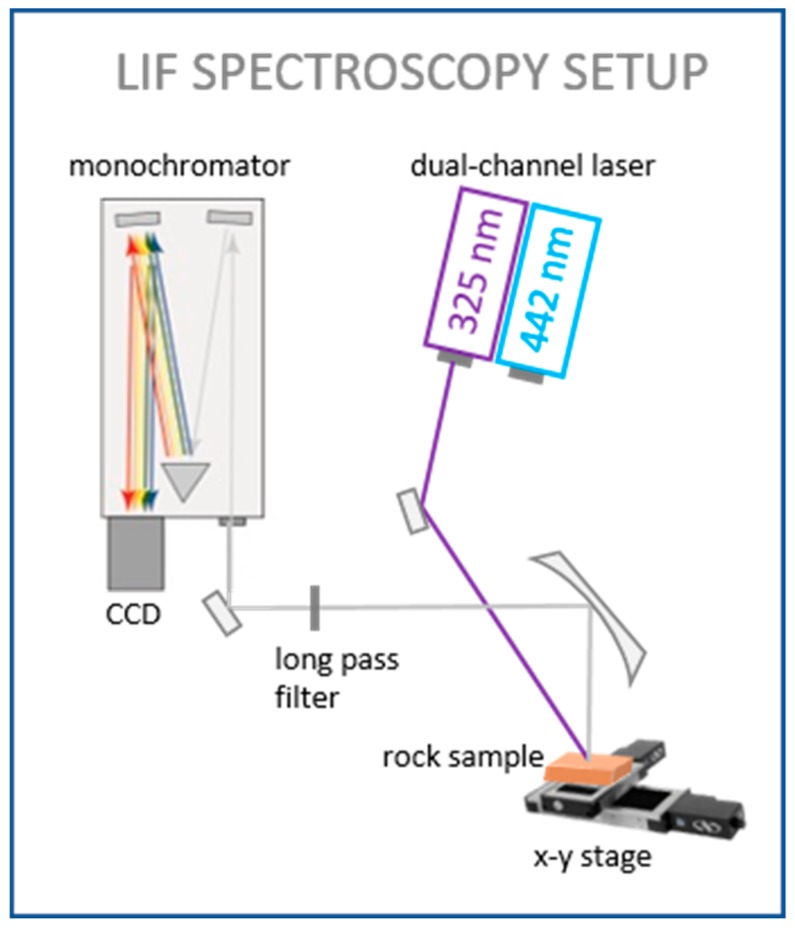
Sketch of setup used for laser-induced fluorescence (LIF) spectroscopy experiments, including laser excitation source, sample on an x-y-stage, monochromator, and a charge-coupled device (CCD) detector.

For the LIF experiments, we used a setting in a darkroom laboratory, as described previously [11]. The samples were exposed to two different laser excitation wavelengths: 325 nm (UV) and 442 nm (blue). The UV and blue lines were generated with a dual-wavelength Kimmon HeCd gas laser and their beam powers were adjusted to 5 mW and 20 mW, respectively, by a neutral density filter. For several excitation tests, an Nd:YAG laser with an excitation wavelength of 532 nm (green) was used. Due to acquisition time limits (see in Section 3.2), we used an unfocused beam on the sample for the mapping experiments (Figure 2). The laser beam was directly guided towards the sample stage, passing two tilted flat mirrors. For several individual point spectra, the beam was guided across an aperture and focused by a parabolic mirror. The Gaussian spot diameters, i.e., where the intensity is >1/e^2^ of the maximum intensity for the focused and unfocused beams and the corresponding excitation power densities, are summarized in Table 1.

In addition to the previous studies [10,11], a remotely controlled, x-y moving stage was employed to generate a LIF map by scanning the sample according to a pre-programmed raster. The micrometer screws of the stage are capable of precise movements in 1-µm steps. Commonly used full-sample scans had a step (pixel) size of 0.5 × 0.5 mm^2^ to 1 × 1 mm^2^, resulting in the acquisition of 800 to 3200 individual luminescence spectra per scan. The sample was fixed on top of the x-y stage, aligned parallel to the moving directions to acquire the full sample area. The outermost millimeter of the rock piece was not scanned to avoid sample edge effects.

Afterwards, the luminescence signal of the sample was collected by a parabolic mirror and guided across two different long pass filters towards an Acton SP2560 triple grating monochromator (300 gr/mm grating, blazed at 750 nm). The wavelength-dispersed photons were finally recorded by a Princeton Instruments SPEC-10:100BR_eXcelon CCD camera, enabling a detection of photons with wavelengths from 340 to 1080 nm. Since the spectrometer images a wavelength range of 170 nm at once, four (blue excitation) to five (UV excitation) maps with different wavelength ranges were acquired and assembled afterwards by post-processing software developed in-house. Data from wavelengths below 360 nm and above 1070 nm were excluded from further analysis because of the low camera sensitivity at the edges of its wavelength range.

The obtained raw luminescence data was corrected for spectral response of the setup and stored as a 3D data cube, in analogy to the hypercube created by the acquisition of hyperspectral absorption data. For 2D visualization, the LIF images showed the intensity distribution in the two spatial dimensions for a given spectral band (grey scale) or in a three-band combination (RGB false color).

### 2.3. Further Experimental Methods for Cross-Validation

Two spectroscopic methods were employed for cross-validation of the 2D LIF mapping results, hyperspectral absorption spectroscopy (HSI) and mineral liberation analysis (MLA). A detailed comparison of different HS imagers for REE detection was given in a previous study [11]. For the experiments presented in this publication, we used an FX 10 push broom scanner from SPECIM, mounted in a SiSuRock drill core scanner framework. The scanner acquires the data line-by-line, while the sample is moving at constant speed. This sensor provides a good spatial resolution, which resulted in our case in a pixel size of 0.5 × 0.5 mm^2^. One spectrum covers a wavelength range from 400 to 1000 nm, equally divided into 224 bands. The spectral resolution of this camera, expressed by the full width at half maximum, is about 5.5 nm. Radiance values were converted to reflectance using a pre-calibrated poly(tetrafluoroethylene) panel (Spectralon SRS-99) with >99% reflectance in the visible-near-infrared range of the electromagnetic spectrum.

The MLA experiments were performed by employing a combination of an FEI Quanta 650F scanning electron microscope with two Bruker Quantax X-Flash 5030 energy-dispersive X-ray spectrometers and the MLA 3.1.4 software package for semi-automated data acquisition [18]. Further, the software was used for data processing and evaluation. The polished and unpolished thin sections were analyzed with a grain-based X-ray mapping (GXMAP) mode. Detailed information about MLA and the offline data processing can be found in [19,20]. For the GXMAP measurements, the electron beam was accelerated by a voltage of 25 kV, resulting in a 10 nA probe current. The mapping was done frame by frame, with each frame having a size of 2000 × 2000 µm^2^ and a frame resolution of 500 × 500 pixels leading to a pixel size of 4 × 4 µm per pixel. The distance between each X-ray measurement was set to 10 pixels and the measurement time to 7 ms.

## 3. Results and Discussion

The sampling of the rock pieces was guided by prior knowledge of the investigated REE deposits obtained by remote sensing techniques, such as satellite and airborne multispectral imaging, and from the knowledge of experienced geologists during the ground survey. The presented samples stem from iron-rich carbonatite trenches (for Lofdal) and calcitic carbonatite trenches (for Epembe). The overall mineralogy of the samples was described by the prospectors as carbonatite-goethite mixtures (for Lofdal) and apatite grains in a calcitic host rock (for Epembe). Geochemical analysis of the whole-rock assays revealed a comparatively high amount of REEs (~0.5% of total rare earth oxide) for both samples. 

### 3.1. LIF Spectroscopy

**Figure 3:**
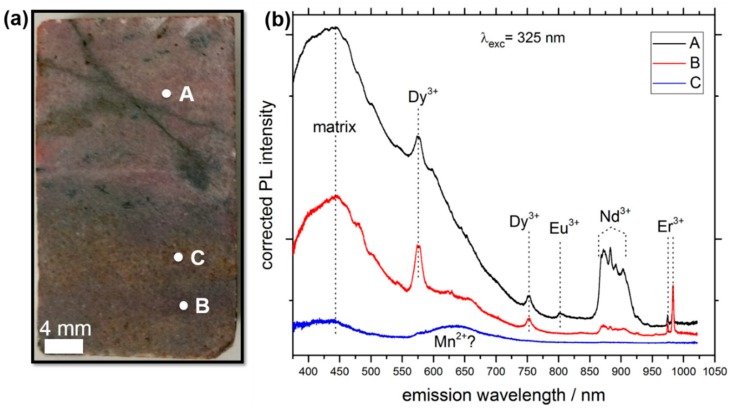
(**a**) RGB image of Lofdal sample marked with three points (spots A, B, and C). (**b**) Focused LIF spectra acquired at the three marked spots. They exhibit different shapes and varying relative intensities of individual peaks (right), which can be attributed to individual REE^3+^ ions. The excitation wavelength of the laser was 325 nm (UV). Note the logarithmic ordinate and the offset for the individual curves for clarity.

As we draw our focus towards complex rocks in the LIF experiments, we observe an expected high variability of both shape of the spectra and position of the REE-related signals across the sample (Figure 3). For example, at an excitation wavelength of 325 nm (UV), distinct sharp features, which are indicators of the presence of REE^3+^ ions, can be seen in the spectra of the spots A and B in Figure 3. The luminescence of REE^3+^ ions exhibit unique features, which are related to their characteristic electronic configuration [21,22]. The sharpness of the emission lines is a result of the screened 4f-4f intraconfigurational transitions, which remain comparably unaffected by the chemical environment [23]. Thus, almost no losses due to multiple phonon emissions occur during the excited state of the REE^3+^ ion, leading to a well-defined emission energy. These sharp lines appear for spots A and B at 575 nm, 750 nm, 800 nm, 978 nm, and in the range of 865–925 nm. Based on the extended research by Gaft et al. [9], in combination with results from our previous studies on REE salts and minerals [10,11], we can attribute these signals to individual REE^3+^ ions, such as Dy^3+^, Eu^3+^, Nd^3+^, and Er^3+^, taking the emission peak wavelength and the shape of the spectra into account.

In contrast to transition metals ions, such as Fe^3+^ or Ti^4+^, the crystal field splitting, induced by the presence of different electrostatic environments for a rare earth ion, is extraordinarily low (~100–200 cm^−1^) [23,24]. A result of this effect is the variable relative intensities of the split emission lines, only if the rare earth ion is hosted in different matrices. Most of the sharp signals exhibit only one peak within the resolution of the obtained spectra. In contrast, the characteristic pattern between 865 nm and 925 nm is most likely solely related to Nd^3+^. It shows a multiplet of emission lines, because of the relatively high crystal field-induced splitting of its ^4^F_3/2_ level, from which the radiation is emitted.

Each spectrum in Figure 3 exhibits several broad features different from the REE signals, spanning a wavelength of a few hundred nanometers. These peaks are more pronounced in the rock spectra than in the REE mineral or REE salt experiments. We attribute these multiple, overlapping emissions to the luminescence of defects in the host rock matrix. Probably, the excitation with high-energy light (UV) activates several transitions of states within the rock-forming crystals. For the detection of REEs in rock samples, these spectral features are less utile, even preventing an identification of REE luminescence signals. Several possible matrix luminescence centers are reported in the literature and are summarized in an extensive review [25]. An explicit interpretation and attribution of these signals in finely-grained rock samples remains uncertain. The origin of this luminescence was not investigated further, but will be part of an additional study.

The comparison of the three different spectra hints to the high compositional variability of the rock sample. While the spectra of spots A and B exhibit REE-related luminescence, none of these features can be seen in the spectrum of spot C, where a broader signal from 570 nm to 700 nm is visible. According to previous studies, this peak can be attributed to the presence of Fe^3+^ or other transition metal ions, such as Mn^2+^ [26,27]. The brown-rusty color of the grains in the RGB image supports this assumption. Although the color of the grains in spots A and C and the shapes of their spectra appear rather similar, small differences in the ratio of the several REE features hint to a distinct composition. Comparing the relative intensities of most of the REE peaks suggests a higher total REE content in spot A (note the logarithmic plotting). Considering the ratio of the Er^3+^-related doublet peaks at 978 nm and 983 nm (corresponding to the ^4^I_11/2_ → ^4^I_15/2_ transition [28]) with respect to the ratio of the other REE^3+^ ion signals, a selective enrichment of erbium in area B is assumed, though, a direct quantification from the emission spectrum is often very challenging and can be erroneous. The absolute luminescence signal from a given solid material also depends on its surface roughness, optical density, and grain size distribution [29]. Non-radiating relaxation of excited states and defects can quench the emissions from luminescence-active materials. Thus, even non-luminescent areas can still contain REEs. To relate the LIF mapping results to the sample chemistry, further probing techniques were used (see Section 3.2).

**Figure 4:**
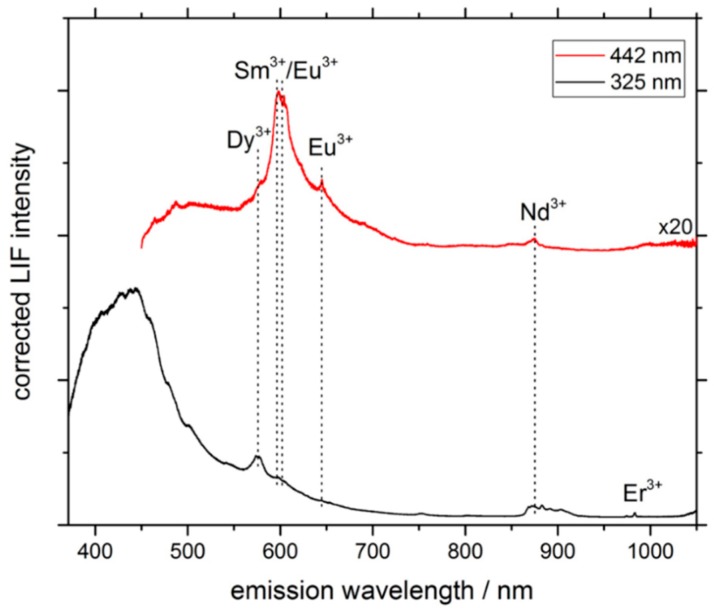
Comparison of LIF spectra from spot A on the Lofdal sample at an excitation wavelength of 325 nm (bottom) and 442 nm (top). Various REE-related signals are marked by dashed lines. Note the artificial enhancement of the spectral intensity for the 442 nm excitation by a factor of 20 and the offset for clarity.

As demonstrated in earlier studies, e.g. [30], the shape of the spectrum and the variety of identifiable REEs change under excitation with different wavelengths (Figure 4). In general, the matrix-related luminescence in the emission wavelength range of 350 nm to 650 nm is significantly stronger under UV excitation conditions, overlapping several REE features. Furthermore, the overall luminescence intensity is clearly reduced using an excitation source at 442 nm, even though the power density of the incident light at the sample is enhanced by a factor of three (see Table 1). Nevertheless, an excitation with 442 nm gives rise to a new REE-related peak, exhibiting a unique shape. For example, a multiplet peak between 580 nm and 630 nm appears, which is attributed to various luminescence centers from different REE^3+^ ions (Dy^3+^, Sm^3+^, and Eu^3+^) [31,32]. A clear correlation between peak position and the corresponding REE cannot be drawn in this wavelength range, since the three REEs are often intermixed in natural samples. In addition, the crystal field-induced splitting and peak shifting of several nanometers complicate a clear identification of the luminescence origin. Employing time-resolved luminescence experiments could help distinguish the signals, since the three luminescences show different decay times [33]. In general, a combination of two (or even three) different excitation wavelengths show the best results for the detectability of a broad range of REEs. While UV excitation is better suited for identification of Er^3+^ and Nd^3+^, a blue laser is more appropriate for the detection of Dy^3+^, Sm^3+^, and Eu^3+^.

**Figure 5:**
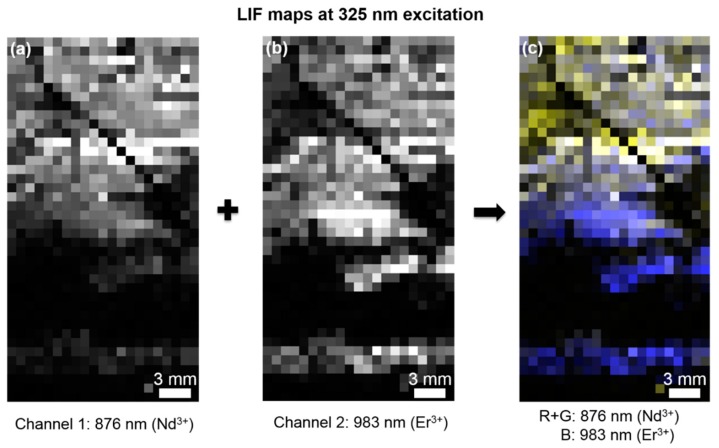
2D LIF maps from the Lofdal sample after excitation with a 325 nm laser at a pixel step size of 1 mm. (**a**,**b**): The grayscale images show the relative intensities of a certain emission wavelength/spectral band, which is related to the occurrence of a specific REE^3+^ ion. (**c**) The normalized combination of both maps results in a false-color RGB map of the sample, where the “red” and the “green” channels are combined. Thus, yellow pixels represent a higher Nd^3+^-related signal and dark blue pixels a higher Er^3+^-related signal. Dark areas do not show any luminescence in the given wavelength ranges.

By using a controllable, micrometer-precise positioning system in x-y directions, we are able to scan the sample in an automated way. Afterwards, the rectangular raster of point measurements is combined to a spatially resolved two-dimensional map of the sample surface by data processing software. Each pixel contains a full emission spectrum of the specific point, resulting in a data structure similar to the one of the hyperspectral imagery. To visualize the acquired 3D data in two dimensions, false-color RGB maps are used, which are created by the combination of two or three different channels, i.e. spectral bands. For example, a false-color LIF map for two REE-related luminescence signals (876 nm [Nd^3+^] and 983 nm [Er^3+^]) after excitation with a 325 nm laser is composed by normalizing the intensities of individual bands to their global maximum value and attributing the band to a certain color in the RGB color space (Figure 5). There, blue pixels signify a higher relative intensity of the luminescence at 983 nm, which hints to higher enrichment of erbium in these regions. The brighter the color, the higher is this intensity. Similarly, yellow (mixed color from red and green) pixels represent areas with higher intensity of the 876 nm luminescence, i.e., hinting at higher occurrences of neodymium. Dark pixels stand for spots where no luminescence in the defined channels was received.

Although the attribution of REE-rich areas to regions with high REE luminescence is challenging (see discussion before), we assume several REE-bearing domains on the Lofdal sample based on the REE luminescence distribution: an Nd^3+^-enriched zone at the upper part and an Er^3+^-enriched zone at the center and at the bottom of the rock piece. In between, there are regions with very low luminescence in the center and at a vein in the upper half. Besides the sensitive detection of several rare earth elements, the LIF mapping at 325 nm excitation contains the additional benefit of localization of the different REE sub-groups. Since most of the REEs occur intermingled within the same mineral phase, single REEs can be regarded as proxies for the REE sub-groups: neodymium for light rare earth elements (LREE) and erbium for heavy rare earth elements (HREE). A LIF map of the Nd^3+^- and Er^3+^-related luminescence enables an extraction of enrichment zones along mineralogical features, such as veins and textures, which is very valuable for exploration of potential REE deposits [34].

**Figure 6:**
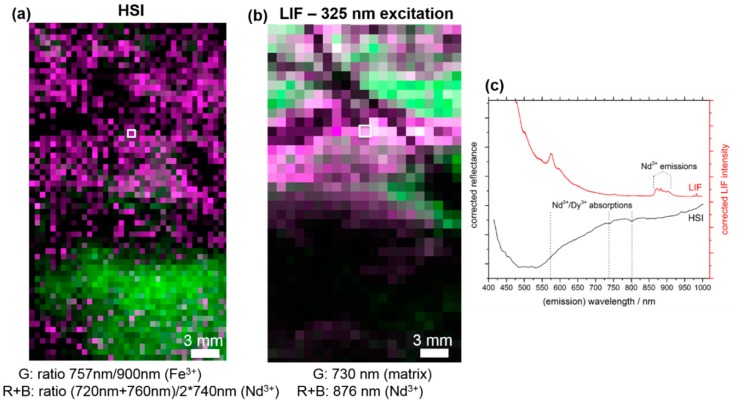
Comparison of false-color maps from the Lofdal sample. (**a**) Hyperspectral imaging (HSI) map (**b**) LIF map under 442 nm excitation. For the HS image visualization, band ratios of Fe^3+^-related (green) and Nd^3+^-related (magenta) absorption features are used. The ratios are calculated by the division of the intensities from the absorption band and from a band that is not affected by this absorption signal. Magenta coloring in the LIF map corresponds to higher relative occurrences of Nd^3+^ ions, while green areas are correlated to the luminescence from the calcitic host rock. (**c**) The comparison of LIF and HSI spectra from one pixel (white squares in (**a**,**b**)) with REE occurrence reveals the differing sensitivity of each method. Dashed lines mark the position of potential REE-related absorption features in the HSI spectrum based on previous studies [36,38].

The ability of localizing luminescence signals in a sample depends on the size of the individual pixel and the resolution of the employed sensor. For applications in the mining industry, such as drill core scanning, the required acquisition time has to be taken into account. We used pixel sizes of 0.5–1 mm^2^, although the resolution of the detection setup and the laser spot would have allowed for 50 µm-wide increments. Still, the acquisition of high-resolution LIF images from a small rock-piece with a pixel size of 0.5 × 0.5 mm^2^ (as seen in Figure 6) takes 30–40 min, which is caused by the comparably low luminescence signal emitted from the samples. For the realization of an in-line drill core scanning sensor, further advances in the sensitivity of the detection system and in the motion control of the positioning system have to be implemented.

### 3.2. Comparison of HSI and MLA

To compare the LIF maps with the results from other 2D imaging techniques, all rock pieces were investigated by HSI as well as MLA. Reflectance spectroscopy in the visible and near-infrared range is already well established for the detection of REE in geological samples [35,36,37]. Especially for REE-rich minerals, such as monazite or bastnaesite, pronounced absorption features, which can be used for the non-invasive detection of several REEs even in sub-% concentrations, have been reported [11,37]. On the other hand, features mainly related to Nd^3+^ and Dy^3+^ dominate the spectra, whereas other REE^3+^ ions exhibit no significant signal. Recently, the superior sensitivity of laser-induced fluorescence spectroscopy for the characterization of REEs in minerals has been reported [11].

Applying LIF spectroscopy to the inhomogeneous Namibian rock samples composed of mainly non-REE-bearing minerals (calcite, goethite), we could confirm their higher REE sensitivity (Figure 6). The HS image is expressed by means of band ratios from Nd^3+^-related absorption features, which is a commonly used method to better visualize absorption signals [37]. The band ratios are calculated by dividing the intensity of the absorption minimum by the intensity of band on a shoulder next to it, which is not affected by the absorption. The green color represents a local enrichment of Fe^3+^ ions, which does not show any luminescence, thus appearing black in the LIF map. In contrast, magenta pixels are related to weak Nd^3+^ absorption features. Although the so-created false-color HS image for three spectral bands exhibits local structures similar to the LIF map, the individual REE signals are more pronounced in the latter image, which is also visualized by the comparison of individual HS and LIF spectra (middle). Whereas no sharp absorption features are visible in the range from 400 nm to 1000 nm, clear REE-related fluorescence signals are observed. Corresponding geochemical analysis from the rock, from which the piece was cut, gave Dy^3+^ amounts of 0.01–0.2 wt%, which are below the detection limit of HSI [11]. Still, these small quantities of REEs could be detected by the LIF measurements, which is the main advantage of using LIF. Magenta pixels (mixed color from red and blue) in the LIF map visualize the occurrence of Nd^3+^-related luminescence, whereas green pixels show a higher relative intensity of matrix emissions.

**Figure 7:**
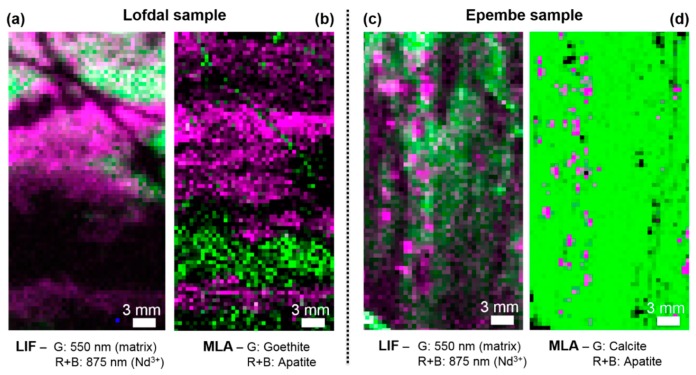
(**a**) 2D LIF map from the Lofdal sample after excitation with a 442 nm laser at a pixel distance of 0.5 mm. (**c**) 2D LIF map under the same conditions for the Epembe sample. (**b**,**d**) For comparison, the corresponding resampled mineral liberation analysis (MLA) maps are shown next to the LIF maps, the depicted mineral phases visualize the occurrence of the main minerals (calcite, goethite, and apatite). Magenta pixels represent the occurrence of REE emissions in the LIF images and the related occurrence of apatite as main mineral phase in the MLA maps.

To validate the obtained results from LIF mapping and correlate the distribution of REE-related signals to mineral phases, we employed MLA on all rock pieces after the LIF measurements took place. Samples from two deposits with differing lithologies were tested to examine the influence of the host rock matrix on the detectability of REEs in geological settings (Figure 7). The shown MLA maps have been resampled to a pixel size of 0.5 mm to meet with the pixel size of the LIF maps. Magenta MLA pixels stand for spots where the mineral apatite is present. Green pixels represent the occurrence of main host rock minerals, such as goethite, FeO(OH), for the Lofdal sample and calcite, CaCO_3_, for the Epembe sample. To avoid an overshadowing of the non-calcitic phases by the predominant calcite phase in the Lofdal sample, the calcite is not shown there. The distribution of REE luminescence matches very well to the occurrence of apatite, suggesting an enrichment of rare earth elements in the apatite grains. This effect is well known from former mineralogical investigations, where apatite, Ca_5_[(F,Cl,OH)|(PO_4_)_3_], was found to incorporate up to 1 wt% of total REEs in its crystal lattice [39,40]. Moreover, no luminescence was obtained from the region where the MLA experiments revealed a goethite vein without any REE-bearing minerals. However, a selective enhancement of the REE luminescence by the apatite host and the quenching of the REE-related luminescence by different minerals could be alternative explanations for the luminescence intensity differences in the various phases. Luminescence quenching in minerals by Fe^2+/3+^ species has been particularly well reported in literature [41,42]. Nevertheless, complementary MLA experiments on the same Fe-rich areas showed no REE occurrences there, suggesting that the quenching effect is not present for our samples. The good match between both distributions demonstrates the ability of the LIF technique to detect such low REE amounts in complex rock samples without damaging the investigated sample.

Furthermore, the lithology variations in both deposits are successfully reproduced by the LIF map, when the correlation between apatite and REE luminescence is assumed. Whereas in the Lofdal sample, the few-micrometer large apatite grains are agglomerated into larger structures and intermingled with the calcite host rock, the apatite is ordered in few-millimeter large mineral aggregations within the calcite for the Epembe deposit. These findings are in good agreement with previous studies on the geology and mineralogy of the Lofdal [14] and the Epembe [16] deposits.

A comparison between HSI, MLA, and LIF, where several metrological parameters for each technique are summarized, is given in Table 2. It should be taken into account that both MLA and LIF are spot-scanning methods, whereas HSI is performed by a line-scanner. In general, MLA proved to be a versatile tool for the validation of LIF imaging results and concatenation of luminescence and local chemistry. Its smaller pixel size allows for a more detailed and direct identification of minerals not only limited to REE-bearing phases. However, 2D LIF spectroscopy is suited for faster and non-invasive detection of REEs and allows for a good differentiation between individual rare earth elements, whereas distinguishing single REEs by the X-ray emission lines used for MLA assignment is challenging. In addition, both the purchase and the maintenance costs are lower for LIF sensors compared to MLA sensors. HSI is the fastest method, but in our setup had the lowest spatial resolution and poorest detection limit for REEs. Both HSI and LIF are in favor of not damaging the sample, in contrast, MLA experiments require small (few cm^2^) samples with flat, polished surfaces, which are coated with graphite and need to be transferred to high vacuum conditions for measurement.

### 3.3. Implications for Mineralogy and Exploration of REEs

As shown in the previous section, 2D mapping by laser-induced fluorescence spectroscopy is a promising technique for identifying individual REEs and revealing their distribution in geological samples. Contrary to classical geochemical methods, such as mass spectrometry and X-ray fluorescence analysis, the samples can be measured faster and in the future possibly in-line, i.e., on a drill core scanner. Furthermore, only limited time is required for the preparation of a flat surface and this surface is not damaged by the laser beam, if a certain (high) limit of excitation power density is not exceeded. On the other hand, only information from the upper micrometers of an opaque sample are collected by LIF spectroscopy, whereas a three-dimensional image of the whole rock piece cannot be extracted. Thus, a careful sample selection and statistical interpolation of the gathered results to the whole rock body are necessary. Further advances in integrating an LIF sensor with other 3D-imaging sensors, such as dual-channel X-ray tomography, could lead to a versatile tool for minimal-invasive detection of REEs in a material stream, regardless of drill-core scanning for exploration or characterization of extracted rock materials in a mine operation. 

However, for a satisfactory analysis of various REEs, more than one excitation wavelength is recommended. From our research, we assume an excitation light with a wavelength of 442 nm as suitable to detect sharp REE luminescence while suppressing overlapping signals from the host rock matrix. By the addition of an excitation with 325 nm, we can clearly examine the occurrence of Nd^3+^ and Er^3+^, which serve as proxies for LREE and HREE, respectively. A LIF map with the relative abundance of LREE and HREE adds a high value to the interpretation of geological structures in the rock samples/drill cores, aiding in the assessment of the REEs’ origin in the host rock (e.g., tracing of magmatic processes or hydrothermal fluids), and thus a better understanding of the whole deposit. In the case of the discussed Namibian deposits, LIF mapping helps us to understand their lithology and their REE enrichment zones. In the example of Lofdal, the sample shows a distinct layering, supporting the theory of a repeated overprint by hydrothermal fluids, which carried the rare earth elements. HREE are locally enriched around iron-rich veins, a state which is in agreement with a previous report on the geochemical dynamics at this deposit [14]. In the case of Epembe, it confirmed apatite as being the major host for LREE enrichment.

Another challenge in the interpretation of REE LIF maps is the correlation of the luminescence intensity to the elemental concentration of the individual REE. Further studies on this specific topic have to be conducted, including comparing LIF spectroscopy with different other techniques, for example Raman spectroscopy for structural characterization of the REE-bearing phase and investigating many samples from various kinds of REE deposits (e.g., ion-adsorption clays, carbonatites, and alkali-pegmatites). We have already performed several experiments for testing the quantification of REEs by LIF, but their results will be presented in a separate publication, due to the extent and complexity of this topic.

## 4. Conclusions

We employed two-dimensional laser-induced fluorescence mapping for the identification of REEs in rock samples. After excitation with blue (442 nm) and UV (325 nm) lasers, we could assign sharp luminescence signals to individual REE^3+^ ions based on previous reports. Using a remote-controlled x-y translation stage, we were able to gather LIF maps of rock pieces from two REE deposits in Namibia, enabling a precise localization of the areas with elevated REE concentrations. The effects of the inhomogeneous rock matrix, complex lithology, and signal dependency on the excitation wavelength have been examined. LIF mapping as a non-invasive imaging technique proved to be a versatile tool for REE characterization, detecting REEs even in a low concentration of 0.02 wt%. Moreover, a comparison with other widely used surface imaging methods, HSI and MLA, revealed the strengths and limitations of LIF spectroscopy. Its superior sensitivity for REEs, comparably high acquisition speed, and low need for sample preparation qualify it for usage as a sensor in the modern mining and exploration industries. The combination of LIF and MLA was especially beneficial, demonstrating, for example, the association of REEs with the apatite grains in Lofdal and Epembe and showing a significant enrichment of HREEs around iron-rich veins for the Lofdal samples, which is in agreement with previous geological studies. This outcome proves that 2D LIF mapping can help geoscientists with the interpretation of geological structures and the dynamics of an REE deposit.

Further investigation using different sensors, such as electron microprobes, could help in establishing a more direct relation between REE concentration and luminescence signals and is subject of another study currently in preparation. Moreover, mechanical and electronic assembling of the main parts, used for our experiments, into a single combined LIF + HSI framework will be the next step for the implementation of an LIF sensor unit, which would benefit the REE exploration and mining industries considerably.

## Figures and Tables

**Table 1:** List of Gaussian spot diameters and resulting average excitation power densities for the employed laser configurations.

Excitation Wavelength	Condition	Spot Diameter/nm	Power Density/(W/cm^2^)
325 nm–UV	focused	0.16	24.9
	unfocused	0.71	1.3
442 nm–blue	focused	0.18	78.6
	unfocused	0.79	4.1

**Table 2:** Comparison of employed 2D imaging techniques for geological samples. The acquisition time is given for the measurements of 4 × 2 cm^2^ rock pieces in steps of 500 µm (HSI + LIF) and 4 µm (MLA), respectively. Detection limits for HSI were taken from [36,43], for MLA calculated from the quantitative analysis, and for LIF from complementary electron microprobe experiments.

Parameter	HSI	MLA	LIF
Acquisition time	~1 s	~10,000 s	~1500 s
Spatial resolution	>300 µm	>2 µm	>50 µm
REE detection Limit	>0.03 wt%	>0.01 wt%	>0.01 wt%
Sample preparation	flat surface preferred	flat surface + C-coating + transfer to high vacuum	flat surface
